# Chemotactic Migration of T Cells towards Dendritic Cells Promotes the Detection of Rare Antigens

**DOI:** 10.1371/journal.pcbi.1002763

**Published:** 2012-11-15

**Authors:** Renske M. A. Vroomans, Athanasius F. M. Marée, Rob J. de Boer, Joost B. Beltman

**Affiliations:** 1 Theoretical Biology, Utrecht University, Utrecht, The Netherlands; 2 Computational and Systems Biology, John Innes Centre, Norwich Research Park, Norwich, United Kingdom; 3 Division of Immunology, The Netherlands Cancer Institute, Amsterdam, The Netherlands; North Carolina State University, United States of America

## Abstract

In many immunological processes chemoattraction is thought to play a role in guiding cells to their sites of action. However, based on *in vivo* two-photon microscopy experiments in the absence of cognate antigen, T cell migration in lymph nodes (LNs) has been roughly described as a random walk. Although it has been shown that dendritic cells (DCs) carrying cognate antigen in some circumstances attract T cells chemotactically, it is currently still unclear whether chemoattraction of T cells towards DCs helps or hampers scanning. Chemoattraction towards DCs could on the one hand help T cells to rapidly find DCs. On the other hand, it could be deleterious if DCs become shielded by a multitude of attracted yet non-specific T cells. Results from a recent simulation study suggested that the deleterious effect dominates. We re-addressed the question whether T cell chemoattraction towards DCs is expected to promote or hamper the detection of rare antigens using the Cellular Potts Model, a formalism that allows for dynamic, flexible cellular shapes and cell migration. Our simulations show that chemoattraction of T cells enhances the DC scanning efficiency, leading to an increased probability that rare antigen-specific T cells find DCs carrying cognate antigen. Desensitization of T cells after contact with a DC further improves the scanning efficiency, yielding an almost threefold enhancement compared to random migration. Moreover, the chemotaxis-driven migration still roughly appears as a random walk, hence fine-tuned analysis of cell tracks will be required to detect chemotaxis within microscopy data.

## Author Summary

T lymphocytes are important actors of the immune system that find and kill infected cells. Before a T cell can mount such an immune response, it has to be activated through contact with a dendritic cell (DC) carrying antigen relevant to the specificity of the T cell receptor. This process typically takes place in secondary lymphoid organs such as lymph nodes and spleen, where DCs scan many T cells at a time. However, the fraction of T cells specific for any antigen is about –, and therefore establishing a contact between a DC carrying cognate antigen and the correct T cells seems quite a challenge. Here, we show with a computational model that despite the presence of millions of competing non-specific T cells, the probability of such a cognate interaction greatly increases when DCs produce a chemokine ligand to attract T cells. The scanning process becomes even more efficient when T cells become insensitive to the chemokine after contacting the DC. These findings oppose the earlier notion that chemoattraction is counterproductive due to blocking of DCs by T cells of irrelevant specificities.

## Introduction

Upon maturation, T lymphocytes continuously circulate in the blood and secondary lymphoid organs such as LNs and spleen. When they encounter dendritic cells (DCs) that present cognate antigen, the T cells become activated and subsequently start to proliferate. Before such an immune response is mounted, the fraction of T cells specific for any antigen is about 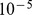
–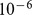 [1]. Because LNs are packed with T cells that have irrelevant specificities, it seems a challenge to establish a contact between a specific T cell and a DC carrying cognate antigen.

Over the last decade, two-photon microscopy (2 PM) experiments applied to living lymphoid tissues have offered a wealth of insight in T cell migration characteristics and T cell-DC interactions in LNs [2], [3]. In the absence of cognate antigen, T cells move at high speeds in an approximately constant direction for up to several minutes, whereas in the long run their migration pattern roughly resembles a random walk [4]–[8]. During their journey through the LN, T cells engage in brief contacts with DCs, lasting a few minutes on average [5], [7], [8]. DCs migrate much more slowly than T cells, and continuously extend and retract long, thin dendrites, thereby greatly increasing the LN volume that they are able to scan [8].

T cell behaviour changes in the presence of activated DCs presenting cognate antigen: After an initial phase of rapid migration and brief contacts, similar to their behaviour in the absence of cognate antigen, T cells form stable contacts with DCs lasting several hours [7]–[9]. Subsequently, T cells resume migration, exhibit signs of activation and start proliferating [8].

In the presence of cognate antigen, it has been shown that ‘licensing’ of DCs by either 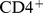 T cells [10], 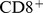 T cells [11] or NKT cells [12] increases their ability to recruit naive 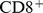 T cells [13], and this is mediated by chemoattractant ligands produced by the licenced DCs [12], [14]. In contrast, 2 PM experiments showed that T cell migration patterns *in vivo* resemble a persistent random walk, suggesting that the migration process does not involve chemotaxis or that chemotaxis only plays a marginal role. Furthermore, it was proposed that the random walk would represent an optimal search strategy [6], [15]. The alternative strategy of chemoattraction of T cells towards DCs was thought to be counterproductive, because nonspecific T cells would also be attracted and subsequently block the DC from scanning other T cells [15]. However, this notion is in conflict with the fact that chemoattraction has been observed *in vivo*, at least when cognate antigen is present, and that such chemoattraction promotes effective cytolytic as well as CD8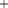 T cell memory responses [12], [14], together suggesting an important functional role for chemotaxis.

The question whether chemoattraction is expected to help or hamper scanning of T cells by DCs was further addressed by Riggs *et al.* [16] using a theoretical framework, i.e. a 2D agent based model of the LN T-cell zone, in which T cells could either migrate in a random fashion, or in addition react chemotactically to a local chemokine gradient around DCs. In those simulations, the presence of chemoattraction led to a reduction of the number of unique T cells contacted per DC and therefore to a less efficient immune response, supporting the view that chemotaxis towards DCs is detrimental. However, this result need not be representative because the model formalism that the authors chose may lead to unrealistic blocking of cell migration in crowded lattices [17]. To alleviate this problem, the authors performed simulations at low cell densities [16]. Nevertheless, when multiple T cells are attracted to the same location via chemotaxis, cell densities become locally high and blocking of cell migration may again arise. Therefore, the model may have generated an answer to the issue that is biologically non-reasonable.

Moreover, it has been pointed out that the typical analysis of T cell migration used in the 2 PM studies, i.e., deriving the type of migration process from a mean (square) displacement plot, is insufficient to distinguish between a random walk and migration amongst several local sources of chemoattractant, as would be the case when multiple DCs in the LN are producing chemoattractant [2]. Taken together, it cannot be excluded yet that chemotaxis enhances the likelihood of establishing interactions between T cells and DCs.

Here, we therefore readdress the question of the expected impact of chemoattraction on T cell scanning by DCs, using the Cellular Potts Model (CPM). We have opted for the CPM because it allows for a mesoscopic description of cell shape, cell migration, chemotaxis and cellular interactions within complex tissue environments [18]. The CPM is a spatial grid-based model formalism that has initially been developed to describe the biophysics of cell sorting due to differential adhesion [19], [20]. Within the formalism, cell motion comes about from the overall minimisation of the energy of deformation and stretching of the cell membrane through stochastic fluctuations, in which the global and local forces upon a cell edge are resolved [21]. For single cells and small tissues, extensions have been made to describe the detailed biophysics and regulation of cortical tension [22], tissue deformation [23], and cell migration and chemotaxis [24], [25]. In contrast, to capture cell migration and chemotaxis within the dynamics of larger and more intricate cell populations, more phenomenological descriptions of those processes have been developed, in which the detailed biophysics were replaced by effective forces along the membrane (for an overview, see [21]). Previously we have shown that such a more phenomenological description of cell migration can be used to realistically and quantitatively capture T cell and DC dynamics within a densely packed LN, with approximately persistent motion on short timescales and random motion on long timescales [26], [27]. We here extend this existing framework with a frequently used CPM extension for chemotaxis [18], [28]–[30] to describe the chemotactic response of T cells to chemokines produced by DCs. Besides the impact of chemoattraction, we here investigate the potential role of T-cell desensitization to the chemoattractant in scanning efficiency. We show that chemoattraction of T cells towards DCs increases the T cell scanning efficiency and thus the probability of T cells to find a rare DC carrying cognate antigen.

## Results

### Model setup

**Figure 1:**
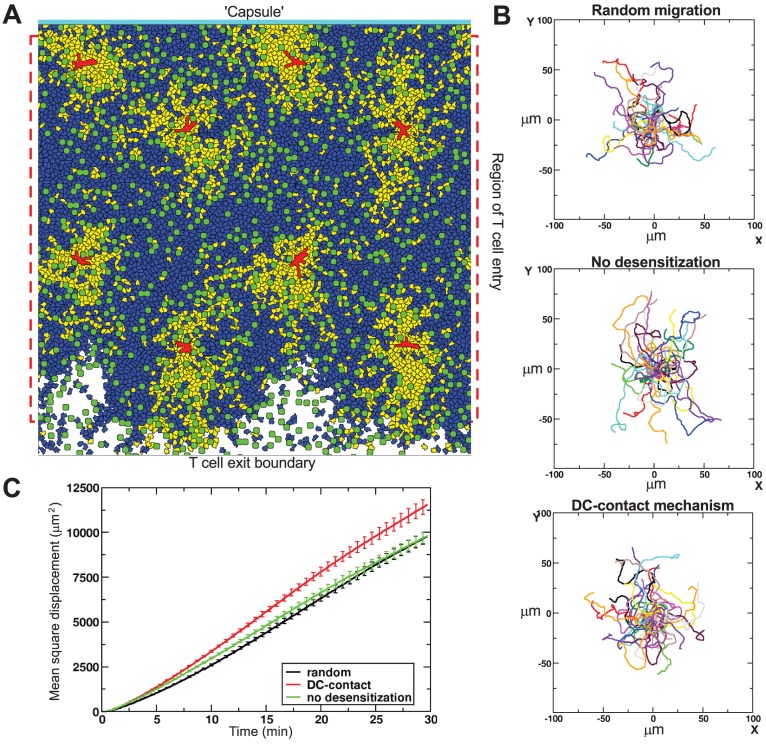
T cell motion in absence and presence of chemotaxis. (**A**) 2D snapshot of a simulation with the DC-contact desensitization mechanism showing sensitive T cells (blue), insensitive T cells (yellow), DCs (red), reticular network (green), and the ‘LN capsule’ at the top (cyan). T cells enter in the region indicated by the red square, and leave at the boundary at the bottom. (**B**) Overlay of T cell tracks from a 10 min period in x and y coordinates after aligning their starting positions to the origin. (**C**) Mean square displacement plots (averages 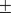 s.e.m.) for the different migration mechanisms. Cellular motility parameters were estimated after combining the data from 10 simulations (see Methods). The estimated motility coefficient, 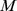, and persistence time, 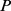, (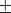 s.e.m.) are: 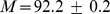 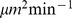 and 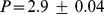 min for random migration; 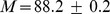 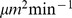, 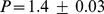 min for the no-desensitization mechanism; and 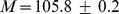 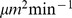, 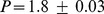 min for the DC-contact mechanism.

We performed most of our simulations using a 2D model of a part of the LN T cell zone around the high endothelial venules (HEVs) through which T cells enter the lymph node, and where T cells and DCs come into contact with each other. Most simulations were done in 2D to be able to more directly compare our results to those of the model by Riggs *et al.* [16], which was also simulated on a 2D lattice. However, we have also performed 3D simulations to confirm our 2D results within a more realistic spatial setting, and to test whether dimensionality plays a role in the relationship between chemotaxis and search efficiency. Our model contained *in silico* T cells (blue and yellow in Figure 1A), DCs (red), reticular network (green) and the capsule (cyan). The latter two elements were included to capture a realistic LN structure.

We modelled entry into the LN by introducing new T cells at random positions in the indicated region in Figure 1A, and exit by allowing T cells to leave the simulations at the bottom. We kept the tissue densely packed with cells, to realistically mimic the situation in LNs. Entry and exit of T cells were balanced such that the number of T cells in the simulation was kept constant, as is approximately the case *in vivo* [31].

In a LN of 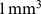, there are roughly one million T cells. Translating this to the volume of our 3D simulations, this would amount to approximately 1000 cells, significantly lower than the 5000 cells used in our simulations. However, because T cell zones largely consist of T cells, the true density of T cells in these zones is closer to our simulated densities than reflected by the average density over the whole LN.

By default, our *in silico* T cells performed a persistent random walk and parameters were tuned such that T cells moved with realistic speeds and motility coefficients [26] (see the section on T cell migration without chemotaxis). In contrast to the T cells, DCs were kept in our simulations at predetermined and more or less fixed mean positions and were distributed approximately evenly throughout the space, as observed *in vivo* [32]. Although some DCs are migratory and carry antigen to the LN, they transfer their antigen to static, lymphoid resident DCs which then activate T cells [33]. Furthermore, it is thought that most DCs die within the LN, and consistent with this, only 0.1% of cells leaving via efferent lymphatic vessels is estimated to be DC. Therefore, we did not include entry and exit of DCs in our model.

The simulated DCs continuously extended and retracted dendrites from their centre of mass (as described previously [27]), giving them a large surface area to be able to contact T cells. DCs could also produce a chemoattractant, which subsequently diffused through the tissue and decayed. The combination of multiple chemokine sources in the field, together with diffusion and decay, generated a complex concentration profile in which the steepness and orientation of the chemokine gradient was highly variable in space.

T cells in our simulations could be in two different states, either sensitive or insensitive to the chemokine gradient created by the DCs. We hypothesized that a natural point in time for T cells to become desensitized would be the moment they establish an interaction with a DC (referred to as the ‘DC-contact’ mechanism). Such a desensitization potentially allows the T cell to leave and other cells to approach the DC. After a recovery period, the length of which we varied, T cells resensitized to the chemokine gradient. To determine the importance of desensitization, we also ran simulations in which T cells did not become insensitive at all (the ‘no-desensitization’ mechanism).

Furthermore, we varied the strength of the reaction of T cells to the chemokine gradient (see Methods). To do so, we varied a T cell parameter (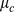) which determined the amplification of the chemokine gradient signal (i.e., 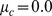 effectively corresponds to random migration, while 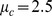 is the strongest chemotactic response we used).

### T cell migration without chemotaxis

Without any obstacles or other cells present, an *in silico* T cell migrates on a short timescale of a few minutes in a more or less straight direction, but in the long run it migrates randomly. When cell density is high, the T cells in the simulation self-organize into large streams of coherently migrating cells, because colliding cells force each other to move into the same direction, and the same holds for streams that bump into each other, until one is left with a single, global stream of cells [21]. Furthermore, our previous simulations predicted that the obstacles formed by RN, DCs and other T cells prevent the formation of such a global stream of T cells, instead triggering the formation of many small, dynamically changing streams, and we confirmed that such microstreams indeed occur *in vivo* by a detailed analysis of 2 PM imaging data [26]. In this study we observe again the formation of such microstreams (Video S1). Moreover, the combination of cell entry at random positions within the tissue and cell exit at the bottom causes a slow overall downward cell flux on top of the chaotic microstreams (see Videos S1 and S2). The downward flux is sufficiently small to not cause any visible bias in the cells' trajectories over short time intervals (of 10 min, see Fig. 1B).

### Impact of chemoattraction on T cell migration

We first set out to examine the effect of chemoattraction on T cell migration without distinguishing between antigen-bearing and non-antigen bearing DCs, or between specific and nonspecific T cells. Thus, all DCs in the simulation produced chemoattractant and all T cells responded in the same manner. This is similar to the simulations by Riggs *et al*. [16], because in those simulations all DCs entering the lattice were quite quickly licensed by 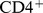 T cells and produced chemokine. Since our first aim was to study whether the negative effects of chemoattraction that were reported by Riggs *et al.*, remain valid in a system where cells can deform, align and squeeze between other cells, we first closely mimicked their situation. Note that, because we did not explicitly model antigen recognition, these simulations would be equivalent to DCs producing chemokine regardless of the presence of cognate antigen. We compared the migration behaviour in simulations without chemoattraction (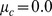, video S1) to simulations with strong chemoattraction (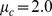, video S2), both for the no-desensitization mechanism and for the DC-contact desensitization mechanism with a fixed recovery time (which was set to 15 min).

The mean speed of T cells migrating without chemotactic cues was tuned to about 11.0 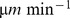, i.e., close to the typical speeds in 2 PM experiments [4], [6]–[8], [34]. The mean speed (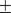 standard deviation within a simulation) slightly increased to 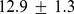 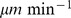 when T cells migrated chemotactically with the no-desensitization mechanism, and to a very similar 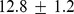 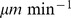 with the DC-contact mechanism. As expected, overlays of normalized cell tracks suggested that in our simulations there was no apparent preferred direction of migration for either randomly migrating cells or chemotactically moving cells (Fig. 1B). The mean square displacement plots varied slightly between the three modes of migration (Fig. 1C). However, the shapes of the curves were very similar, and therefore it is unlikely that chemotactic attraction of T cells towards DCs can be detected with a mean square displacement plot based upon 2 PM imaging data, confirming the suggestion of Germain *et al.* [2].

### Detection of chemoattraction by measuring the angle of migration towards DCs

**Figure 2:**
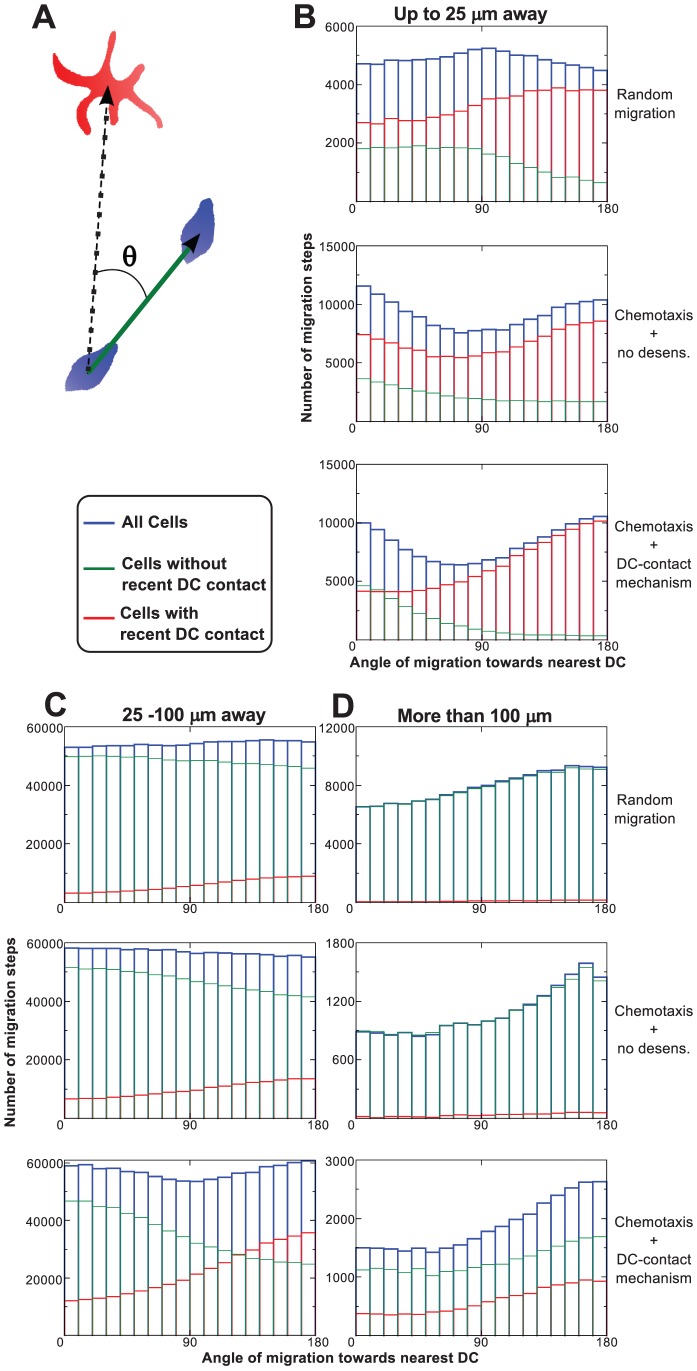
Chemoattraction can be detected using angle measurements. (**A**) The green arrow represents the movement of a T cell over a short time interval, derived from its locations at two consecutive measurement time points. The black dashed arrow is the vector from the centre of mass of the T cell towards the centre of mass of the nearest dendritic cell. The migration angle 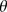 may vary from 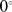 (exactly towards the nearest DC) to 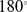 (exactly away from the nearest DC). We performed these measurements throughout the simulation for every T cell and DC (all DCs produce chemokine). (**B**–**D**) Histograms of migration angles 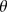, for various distances from a nearest DC. The blue histograms show all movement steps within a representative simulation; the red histograms show only those steps that were made by cells which came in contact with a DC during the last 15 minutes (in the case of the DC-contact method, these cells are insensitive); and the green histograms show the steps made by cells that did not recently contact a DC (which are the sensitive cells in the DC contact method). The simulations have been done with half the T cell density used for Fig. 1 (about 3300 T cells in the entire field), in order to reduce the effect of the downward flow.

We next investigated whether it is possible to distinguish between random migration and chemoattraction to DCs from the simulated cell tracks. To do so, we determined the angle (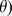 between a vector in the direction of T cell migration as measured between two consecutive time points and a vector pointing from the T cell towards the nearest DC (Fig. 2A). For random migration in two dimensions, every angle is expected to occur with equal probability, but when T cells are attracted towards DCs, acute angles (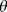 less than 90 degrees, see Fig. 2A) are expected to occur most frequently [35].

Without chemotaxis, the overall distribution of those migration angles for *in silico* T cells that were less than 100 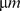 away from their nearest DC was close to uniform (Fig. 2B,C). In the presence of strong chemotaxis, close to DCs we indeed more often observed acute angles compared to intermediate angles, both for the DC-contact mechanism and the no-desensitization mechanism, indicating that attraction of T cells towards DCs could be detected in our simulations.

Surprisingly, when T cells migrated chemotactically, close to DCs also obtuse angles (more than 90 degrees) were observed more frequently than intermediate angles, suggesting that a subpopulation of the T cells was effectively repelled from the DCs. This phenomenon was actually a consequence of spatial exclusion: for every T cell that approached a DC another T cell had to make room for it by leaving the area. In a more detailed analysis we distinguished between T cells that had recently contacted a DC and the remaining T cells. (Note that in the DC-contact method this corresponded to the insensitive and sensitive cells, respectively.) This analysis showed that both in the simulations with and without chemotaxis, the cells which were in recent contact with a DC were the ones that were effectively being repelled, although the effect became more pronounced with chemotaxis (Fig. 2B,C). In fact, cells with recent DC contact seemed to be part of a micro-stream of T cells moving away from the DC (Video S2), suggesting that the process was similar to convective flow.

T cells which were at larger distances from the nearest DC were often at similar distances from other DCs as well, which could strongly influence their migratory behaviour, resulting in a drop in the migration bias towards that nearest DC. When T cells were more than 100 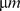 away from the nearest DC, not only the bias towards the nearest DC had disappeared, but T cells were even preferentially moving away from the nearest DC (Fig. 2D). This reversal of migration bias was due to the slow background flow of cells from top to bottom: all cells within the simulated tissue are slowly pushed downward by each other, which explains the migration away from the nearest DC.

These results show that measuring migration angles of T cells that are close to a DC may allow to distinguish between random migration and chemoattraction towards DCs using 2 PM imaging data acquired in the absence of cognate antigen. (Note that it has already been successfully applied to show that 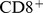 T cells are chemotactically attracted towards licenced DCs [36].)

### The influence of chemoattraction on T cell scanning by DCs

We showed above that current 2 PM data are consistent with the notion of chemotactic attraction towards DCs. In light of the ongoing debate on the importance of chemoattraction (see [2]), we next used our *in silico* environment to examine whether chemotaxis enhances T cell scanning by DCs, compared to randomly migrating T cells. This was done by varying the strength of chemoattraction of T cells towards DCs (i.e., 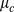, see Methods), and measuring the scanning rate.

**Figure 3:**
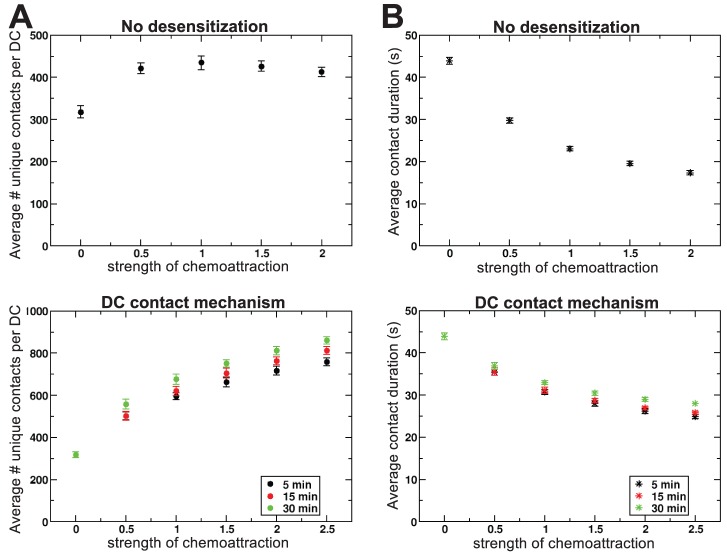
Chemoattraction promotes T cell scanning by DCs. (**A**) The mean number of unique contacts between T cells and DCs and (**B**) the mean duration of contacts, both as a function of the strength of chemoattraction (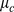). Symbols represent averages over 20 one-hour simulations, and error bars represent standard error of mean between simulations. Legends indicate colour code for the recovery times used in the simulations.

Focussing on unique contacts only (i.e., contacts established with T cells which had not been seen before by this DC), we found that DCs scan about 300 randomly migrating T cells per hour (Fig. 3A, top). With the no-desensitization mechanism, we found a substantial, 40% increase in the number of unique T cells that contacted a DC at the highest chemotactic strength that we simulated. The total number of contacts per DC (i.e., when repeated T cell contacts to the same DC were counted as well) also increased strongly (Fig. S1). Because many more cells visited each DC within the same timespan, the interactions between T cells and DCs lasted on average shorter for strong chemoattraction than for random migration (Fig. 3B, top), indicating that there was fierce competition between T cells for contacting DCs.

For the DC-contact mechanism we found that chemoattraction very strongly increased the number of unique contacts between T cells and DCs, with a threefold higher number of unique contacts at maximal chemotaxis strength compared to random migration (Fig. 3A, bottom). Although the average contact duration also decreased in these simulations, the effect was less strong than for the no-desensitization mechanism (Fig. 3B, compare top with bottom), suggesting that desensitization reduced competition between T cells around the DC. Furthermore, the reduction in the contact duration was slightly weaker for longer recovery times, despite the fact that longer recovery times led to the scanning of more unique T cells than shorter recovery times (Fig. 3A). This was because a sufficiently long recovery time allowed insensitive cells to ‘escape’ from the chemokine attraction field of a recently contacted DC and subsequently contact other DCs. Moreover, longer recovery times allowed for longer T cell-DC interactions because competition around DCs was reduced. In short, the DC-contact mechanism caused a strong coordination in T-cell movement (Fig. 2), leading to a higher motility coefficient (Fig. 1) and allowing for cells to escape the chemoattractant field around the DC, all together causing the high scanning efficiency of this mechanism.

In contrast, Riggs *et al.* [16] used a different method of desensitization. To make a better matching comparison, we also tested this alternative mechanism, even though it might be biologically less reasonable. This method decoupled T-DC contact from desensitization, by letting T cells become insensitive after having been in contact with the chemokine for a certain fixed time period (the desensitization time, see Methods). In these simulations we observed an optimal duration of the desensitization time, which depended on the concentration threshold at which the T cell started to sense the chemokine gradient (Fig. S2). The higher this threshold, i.e., the closer to the DC the T cell had to be to sense the gradient, the shorter this desensitization time had to be in order to achieve efficient scanning. This is because scanning is most efficient when desensitization occurs around the time the T cell gets into contact with the DC. Moreover, it was less efficient to have an overly short desensitization time (e.g., 1 min) than to have an overly long desensitization time (e.g., 30 min; Fig. S2). This confirmed our finding that chemotaxis always increases the efficiency of DC scanning, while it is an even more efficient strategy to become insensitive soon after being in contact with a DC. Regardless of the exact mechanism of chemoattraction used, we consistently observed more efficient T cell scanning for chemoattraction towards DCs compared to random migration. Therefore, these results clearly showed that chemoattraction is expected to promote T cell scanning by DCs.

### Effect of chemotaxis on residence time and long-term scanning rates

**Figure 4:**
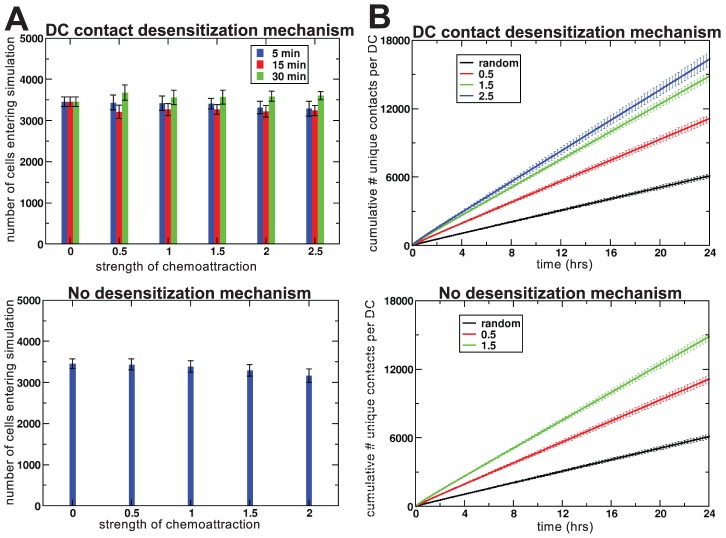
T cell flux through the tissue and long-term scanning efficiency. (**A**) Mean number of cells (for 20 simulations) entering the field during the one-hour simulations, plotted as a function of the strength of chemoattraction (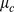). Legend indicates colour code for the distinct recovery times used. (**B**) Unique T cell-DC contacts as a function of time, averaged over 10 simulations. Legend indicates colour code for the distinct values of 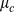 used. Error bars represent standard error of mean.

Because in our model the entry of new cells into the tissue occurred only when other cells left the tissue, chemoattraction might decrease the influx of new cells by keeping cells within the tissue that otherwise would have left. Alternatively, chemoattraction could increase the influx because of the larger motility coefficient. In the latter case, our observation of chemoattraction increasing the T cell scanning efficiency by DCs might be due to an increased influx of new T cells instead of a more effective search amongst the cells that were already locally present. However, the number of cells entering the simulation hardly changed in the presence of chemoattraction (Fig. 4A), so this scenario could be excluded. About half the T cell population left our simulated space over the timespan of an hour (Fig. 4A), and the average residence time of a T cell in our simulations was approximately 1.5 hours.

To investigate whether chemotaxis remains efficient over timescales longer than one hour (our typical simulation time), we also ran some simulations lasting for 24 hours. Figure 4B shows that the number of unique first contacts increased linearly with time, demonstrating that scanning in the presence of chemoattraction remained efficient at long timescales, during which numerous cells entered and left the simulated area. In conclusion, independent of the small variability in the entry rate of T cells, chemotaxis leads to efficient T cell scanning by DCs at both short and long timescales. Our conjecture is therefore that the negative effects of chemotaxis reported by Riggs *et al.* [16] were due to their model formalism. However, it is not immediately clear whether the positive effects we observed in our model still hold in the context of rare cognate antigen, and we turned to a more realistic, 3D version of our model to investigate this.

### Finding a rare antigen-bearing DC

**Figure 5:**
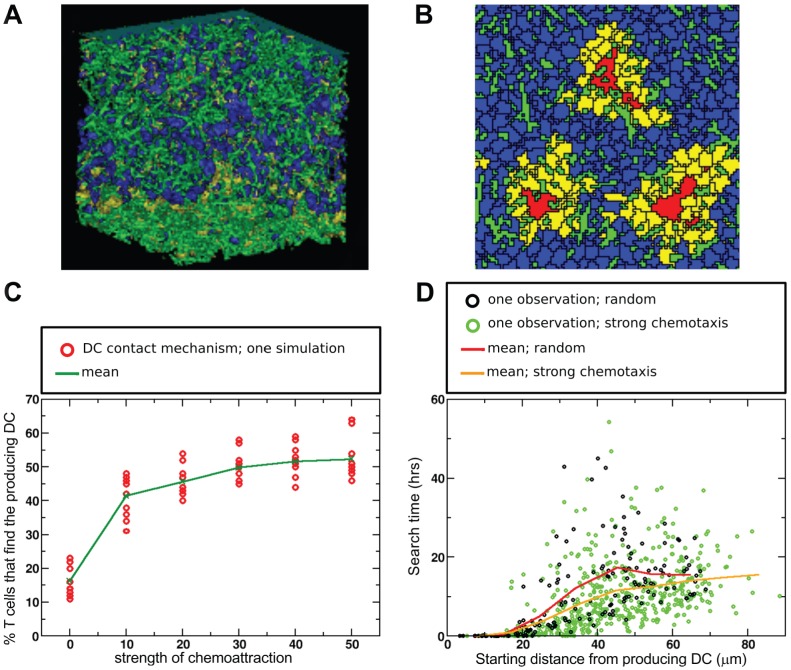
Chemoattraction promotes establishment of cognate T-DC interactions. (**A**) 3D picture of the field. Elements are coloured as in Figure 1: sensitive T cells (blue), insensitive T cells (yellow), DCs (red, but blocked from view by the surrounding T cells), reticular network (green), and the ‘LN capsule’ at the top (cyan). (**B**) Cross section through the field at a position where several DCs can be seen. (**C**) Percentage of the selected 100 T cells in our simulations that establish contact with a single cognate DC producing chemokine. For each value of 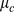 ten simulations were performed per desensitization mechanism (each circle represents the outcome of one simulation and crosses with connecting lines represent means from multiple simulations). The desensitization time for the DC-contact mechanism was set to 15 min. (**D**) The time it took for T cells to find the chemoattracting DC as a function of the starting distance from that DC. Circles represent measurements from individual T cells and lines denote the mean search time per distance bin of 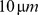. Note that the means at large distances are based on few datapoints only.

We next addressed the question whether there are differences in the efficacy of scanning with chemotaxis compared to random migration when the DCs producing chemokine are rare. To capture this situation, we allowed only a single DC (located centrally in the field) to produce chemokine. To increase the realism of our simulations, we simulated a 3D LN tissue with a similar layout as used for the 2D simulations (Fig. 5A,B). Because it is unlikely that there is a difference in cellular properties (migratory or otherwise) between antigen-specific T cells and nonspecific T cells prior to contact with the cognate DC, we scored for a large number of T cells (representing antigen-specific T cells) in each simulation whether they were able to find the DC bearing cognate antigen. Specifically, we followed 100 T cells per simulation entering the tissue after an initialization period. For each of these T cells we recorded whether they managed to come into contact with the antigen-bearing DC or left the tissue without such a contact. The output of each simulation was the percentage of T cells finding the single chemoattracting DC.

Using the DC-contact mechanism, the percentage of T cells arriving at the chemoattracting DC increased markedly (more than 3-fold) with increasing chemotaxis strength (Fig. 5C).

We also investigated the time it took until T cells found the DC presenting cognate antigen, as well as their starting distance to the DC in case they managed to find it. As shown in figure 5D, it took T cells slightly less time to find the cognate DC in the presence of chemoattraction compared to random migration. Thus, chemoattraction modestly speeds up the search process for T cells that successfully find the chemokine-producing DC, and greatly increases the probability of establising cognate interactions between T cells and DCs.

Random search processes depend strongly on the dimensions of the space considered, because cells are more likely to revisit previously searched regions in 2D than in 3D, which makes a 2D random search less effective [37]. Additionally, crowding effects due to chemoattraction might be less prominent in 3D than in 2D, because there exist more ‘escape’ routes in 3D. Therefore, we performed similar simulations with a single attracting DC in 2D.

Consistent with the 3D simulations, the the effects of chemotaxis on the efficiency of scanning increased very strongly in 2D (Fig. S3 A). However, in contrast to the 3D simulations, chemotaxis did not speed up the search process (Fig. S3 B). Despite these small differences, it is clear that in both 2D and 3D the scanning efficiency is enhanced by chemotaxis of T cells towards dendritic cells, thus contributing to an effective and timely immune response.

## Discussion

2 PM imaging experiments have shown that T cell migration in LNs roughly conforms to a persistent random walk [4]–[8]. However, subtle chemotactic migration could well be hidden in such data. For example, we recently discovered a small yet biologically relevant directed migration component amongst germinal centre B cells [38], for which migration had earlier been described as random [39]. Similarly, Textor *et al.* [40] recently showed that a uniform (e.g., from LN ingress to egress points) chemotactic migration component of considerable size could be present in LN T cells in the absence of cognate antigen. Hence, the same is likely true for a chemoattraction component towards DCs.

Here, we used computational modelling to address the question whether chemoattraction of T cells towards DCs is expected to promote or to hamper the scanning efficiency of DCs. Our simulations showed that, in the absence of cognate antigen, chemoattraction towards DCs enhanced their T cell scanning efficiency about three-fold compared to random migration. Furthermore, when T cells in our simulations had to find a single, centrally located DC that produced a chemoattractant, this search was most successful when there was both strong chemoattraction towards that DC and desensitization upon arrival of a T cell. From these simulations we also learned that the efficiency of the search mechanism hardly depends on the dimensionality of the tissue and is thus very well suited for the complex LN environment.

Consistent with our finding that chemoattraction of T cells towards DCs is more efficient than a random search process, 2 PM imaging experiments have revealed that chemotaxis towards licenced DCs indeed occurs *in vivo* [11], [12], [36]. Chemotactic migration amongst multiple local sources of attractant is not detectable by a mean (square) displacement analysis [2], [35], which we here confirm for our simulation data. Rather, measurements of T cell migration angles relative to the vector towards a nearby DC can be used to detect potential chemoattraction (note that this is also how chemotaxis towards licenced DCs was demonstrated experimentally [36]). It might be possible to make such an angle analysis more powerful by distinguishing between cells that had a recent contact and those that did not.

The angle measurements of the T cells in our simulations offer an explanation for why especially desensitization upon DC contact renders a very efficient search process: the migration pattern of cells that just desensitized gave the impression that these cells are moving away chemotactically from the DC (Fig. 2). However, we did not include such chemorepulsion in our model and the pattern must therefore purely result from the pushing away of insensitive T cells by sensitive T cells. Insensitive cells are pushed away more easily than sensitive cells. Sensitive cells have a bias towards the DCs which the insensitive cells do not have. Therefore, when a sensitive cell on its way to a DC collides with an insensitive cell, it is likely that the insensitive cell is pushed into a different direction of migration while the sensitive cell continues, eventually causing the formation of a small stream of sensitive cells moving towards the DC. Furthermore, when those sensitive cells reach the DC and become insensitive cells, they persist in migrating in the direction into which they are pushed by the sensitive cells behind them, causing them to form a stream that moves away from the DC. In this way around the DCs convective streams are formed, with sensitive cells moving towards the DC at one spot and insensitive cells moving away from the DC at another spot, analogous to the organization of crowds of pedestrians in a busy city centre or subway [41]. This process reduces competition near DCs when the DC-contact mechanism is used, allowing for longer contacts than in the simulations without desensitization. Furthermore, the efficient displacement of insensitive T cells away from DCs that they had already contacted allows these cells to swiftly establish contact with other DCs, thus resulting in efficient T cell scanning.

Although desensitization of individual receptors has been shown to occur in leukocytes [42], [43], it is currently not known whether T cells can desensitize to a chemokine gradient. The presence of such desensitization upon DC contact would require fast signalling between T cells and DCs. Indeed, signals between T cells and DCs may be transferred in the course of seconds [44] and therefore it is possible that desensitization occurs even for brief T-DC contacts. An alternative could be that T cells become sensitive to other chemokines after contact with a DC, allowing them to move away from that DC. However, this seems unlikely because each DC should then produce a different chemokine. If alternatively the other chemokine is produced by an entity outside the T cell zone, T cells would leave the T cell zone soon after their contact with the first DC, and would likely miss DCs carrying cognate antigen, although such a mechanism could make sense after a T cell made contact with a DC carrying cognate antigen. Thus, although there is currently little experimental evidence for desensitization and resensitization, i.e., the loss and recovery of sensitivity to the chemokine gradient, our results suggest that loss and recovery are expected to lead to more efficient scanning. This is because it allows T cells to more easily reach DCs different from those already seen, and as such have a higher probability to find rare antigens. Once a T cell has recognized cognate antigen presented by a DC, other pathways need to be induced to stabilize the interaction. This likely does involve other chemokines [45] as well as formation of an immunological synapse [46].

Contrary to our findings, earlier simulations using a 2D agent-based model of T-DC interactions in the presence of a chemoattractant gradient suggested that chemotaxis hampers the scanning efficiency of DCs [16]. The negative effect of chemoattraction in those simulations was a consequence of chemotactic T cells blocking access to the DCs [16]. These results, however, may stem from the fact that agent based models have the intrinsic property that cells cannot move into lattice positions that are already occupied. Therefore, in these models cell migration cannot be properly captured when cell density is high without the use of additional assumptions: the *in silico* cells cannot squeeze past each other [17] and cannot push each other, whereas 2 PM imaging studies have shown that T cells in the LN are highly flexible, readily change their shape and migrate rapidly despite the densely packed environment [4]. In the simulations by Riggs *et al.* [16] the authors attempted to alleviate this problem by reducing the T cell density to below-physiological values. However, in the scenario with chemoattraction of T cells towards DCs, T cell densities would still become locally high, thereby reintroducing the problem of blockage. Thus, their result that chemoattraction reduces the number of unique T cells that are scanned by DCs seems to be a consequence of the model formalism. It would be interesting to attempt to solve the problem of blockage in such agent-based models on a lattice by either allowing two cells to temporarily occupy the same lattice site such that they can pass each other [47], or to allow for swapping of cells [48], [49]. (Note that ‘convective flow’ as we observed is unlikely to occur in such simulations, because cells would still not be able to push each other.) Another CA-based model, which combines persistent motion and chemotaxis, has been proposed for for B cell activation in the lymph node [50].

As an alternative approach to reinvestigate the question whether chemoattraction of T cells towards DCs is expected to promote or obstruct scanning, we employed the CPM formalism [19], [20]. Using this formalism, we were able to show that even in a densely packed field combined with chemoattraction of cells towards DCs, no blocking occurs and T cells can keep on migrating by forming a convective flow around the DCs. We conjecture that the difference in model behaviours is because the CPM is able to properly describe the shape and flexibility of biological cells as well as their interactions with other cells within a densely packed area (e.g., [26], [27]). In the CPM cells are represented by multiple lattice sites, allowing them to undergo complex shape changes. Combined with the ability to push and pull each other, microstreams are generated that organize the circulation of T cells near DCs.

For these simulations, we chose not to explicitly model the subcellular processes that play a role in chemotaxis. Instead, we applied a phenomenological shortcut to capture these processes, which allowed us to study the consequences of many interacting cells responding chemotactically to a very complex and dynamically changing chemotactic field. Our approach was further simplified in the sense that we did not study the disturbance of the chemokine gradients in the LN by the migrating cells themselves. Indeed, it is difficult to imagine how a gradient could be maintained in the presence of numerous, frantically moving T cells. As a possible mechanism, it has been proposed that secreted chemokines may be rapidly immobilized on the reticular network [51], forming a gradient for T cells to follow. Although we did not study this in our CPM simulations, we expect that such a role of the network would give similar results as the scenario we considered here. In conclusion, we have shown that chemoattraction of T cells towards DCs is expected to increase the efficiency of T cell scanning by DCs, thereby greatly contributing to a timely immune response.

## Methods

### Model description

In the CPM model formalism [19], [20], cells consist of multiple lattice sites (with 2D coordinates i and j, or 3D coordinates i, j and k), and have a type 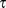 and identification number 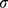. Lattice sites of the cell in contact with the surrounding environment (other cells, medium, RN) have a surface energy 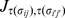 which depends on the type of the cell (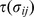) and that of its neighbour (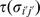). Cells are assumed to minimize their surface energy while at the same time trying to maintain their volume at a target value 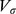. During updates of the lattice, the probability of a randomly chosen neighbour to extend into the site under consideration depends on the so-called Hamiltonian (given for the 2D case only):
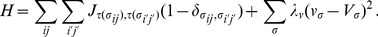
(1)


The first term represents the sum of all surface energies 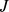, where 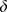 is the Kronecker delta and 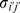 sums over all 8 neighbours in the 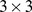 neighbourhood. The second term keeps the actual volume 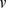 close to the target volume 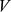, where 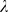 is the inelasticity of cells. The probability that a neighbouring site extends into the lattice site under consideration is 1 if 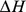 ¡ 0, and e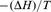 otherwise, where 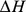 is the change in the Hamiltonian due to the considered modification, and T represents the membrane fluctuation amplitude of cells. The model was implemented using the C programming language, and the cell migration measurements were performed using customized Perl scripts.

### T cell motility and chemotaxis

*In silico* T cells exhibit a target direction, and extension of lattice sites into that direction occurs with a higher probability than extension into the opposite direction. This was incorporated by extending 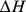 for T cells:
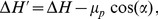
(2)where 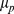 is the ‘directional propensity’ of cells, and 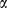 is the angle between the target direction and the displacement vector under consideration (i.e., the vector given by the mean position of the cell and the coordinates of the position to be modified). The target direction is updated every 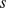 seconds according to the actual displacement of the cell.

Apart from this baseline motility that gives rise to a persistent random walk [26], T cells in our simulations respond chemotactically along a local gradient. Extension into sites with a high chemokine concentration is favoured, and this depends on local subcellular chemokine concentration differences. This is implemented into 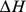 as follows (shown for the 2D case):
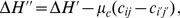
(3)where 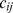 is the chemokine concentration at the lattice site under consideration and 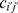 is the concentration at the neighbouring site that attempts to extend. An increase in 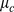 causes cells to react more strongly to a gradient. However, there is a limit to the extent to which 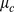 can be increased, because at some point the term driving chemotaxis becomes stronger than the volume conservation term, which can cause T cells that are pushed against DCs by other T cells to reduce their volume to zero and ‘die’. In all simulations, we keep 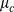 below the point where this non-biological behaviour occurs.

In some simulations, T cells remain sensitive to the chemokine gradient for the entire duration of their stay in the simulation, which we call the no-desensitization mechanism. In simulations with T cells that are insensitive to the chemokine gradient, the second extension of 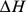 (equation (3)) is only taken into account for the sensitive cells. We implemented two manners in which T cells could become insensitive, referred to as the DC-contact method and the gradient-contact method. In the DC-contact method, T cells become insensitive immediately upon contact with a DC. In that case, they remain insensitive to the chemokine gradient for the duration of a ‘recovery period’ (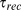) counting from the time of first contact. The gradient-contact method is independent of contact with DCs. Instead, T cells become insensitive after a ‘desensitization time’, i.e., they desensitize 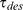 minutes after the first chemotactic response, which is initiated when the cell has sensed a chemokine concentration above a threshold value 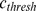. They subsequently resensitize after a recovery period of 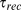 minutes.

### DCs

As described earlier [27], DC dendrites are modelled by defining multiple actin bundles protruding from positions within the DC and retracting after a pre-set time period. In brief, the bundles grow in a random, straight direction, provided that the sites to be copied into belong to the DC. Protrusion of the membrane is achieved by increasing the likelihood of DC membrane elements copying into positions adjacent to a bundle (in that case 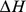 is decreased with 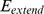). To prevent breaking of dendrites, membrane elements at or adjacent to a bundle cannot be copied into. When bundles cannot extend for 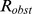 timesteps due to obstacles or chance processes, they retract. Otherwise, the bundle retracts after a maximum of 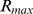 timesteps. Retraction occurs with a probability 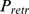 per time step, and as soon as a bundle has completely retracted, a new bundle starts to grow out in a random direction. Each DC has 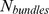 dendrites at a time.

*In silico* DCs produce chemokine, and the chemokine concentration *c* is followed over time in each lattice site of the grid (measured in arbitrary units). The chemokine diffuses with a diffusion coefficient 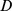, irrespective of the occupancy of lattice sites by cells, RN or extracellular medium. This gives the following equation:
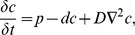
in which the chemokine production term 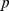 is limited to the DCs only, while the decay, given by 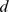, takes place all over the tissue.

### Default model parameters

Unless mentioned otherwise, we use the default parameters defined here. Our 2D model consists of a wrapped square of 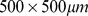, and the (wrapped) 3D model space is 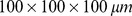. One site of the lattice represents 1 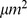 (or 1 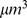).

Cell flow across the upper boundary is blocked by an obstacle representing the lymph node capsule, which spans the width of the field (Fig. 1A). When T cells touch the lower boundary their target area is set to zero so that they shrink and leave the simulation. When the cell has disappeared, a new T cell immediately enters at a random position. In this manner, the number of cells in the simulation remains constant, and there is a weak flow of T cells downward. The number of T cells in the 2D field is close to 6700, and close to 5000 in the 3D field, resulting in fields that are nearly completely packed with cells and RN. Small changes in T cell density do not have a qualitative effect on the T cell scanning efficiency of DCs. However, at very low densities chemoattraction retains T cells in the field, which leads to a decreased long-term scanning efficiency compared to random migration.

We previously found that in the absence of obstacles, densely packed T cells in CPM simulations tend to form massive streams [26], which are not observed in a real lymph node. In the presence of an *in silico* reticular network the streams turn into more realistic local, highly turbulent microstreams. We therefore included a representation of the reticular network by incorporating 900 randomly placed circular objects with a radius of 4 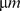 into the 2D field (about 18% of the space) and 3000 randomly oriented rods with a 1 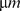 radius and a length of 20 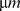 into the 3D field (about 17% of the space).

T cells are initialized at random positions, whereas DCs are placed according to predefined coordinates that are the same for every simulation (Fig. 1A). During a simulation DCs are not allowed to move large distances, which is achieved by having their dendrites grow out from a 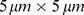 block around the initial position of the DC. All cells are initialized as a 9 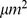 block in 2D or a 27 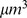 block in 3D, after which they quickly grow out to their target area (30 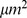 or 150 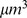 for T cells and 100 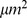 or 1400 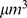 for DCs).

T cells are considered to have a slight preference to adhere to DCs, and there is no differential adhesion between other cell types. Preferential adhesion is implemented as a negative surface tension (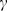) between cell types x and y, and is calculated from the surface energies as follows: 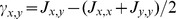. The default surface energy and surface tension parameters are shown in Table 1.

**Table 1:** Default surface energies and surface tensions for both 2D and 3D simulations.

	Tcell	DC	RN	Capsule	ECM
**Tcell**	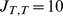	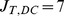	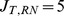	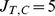	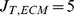
**DC**	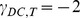	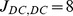	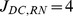	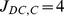	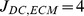
**RN**	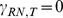	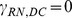	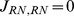	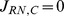	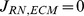
**Capsule**	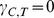	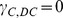	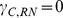	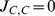	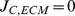
**ECM**	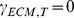	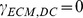	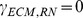	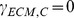	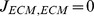

Other default parameters are 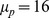, 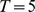, 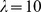, 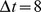, 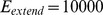, 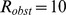, 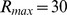, 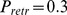, 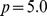, 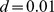, 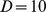 for 2D simulations; and 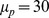, 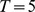, 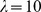, 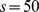, 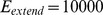, 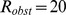, 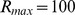, 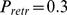, 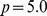, 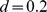 and 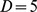 for 3D simulations.

### Simulation measurements

After one Monte Carlo timestep, all sites in the lattice have been considered for updating, which corresponds to 1 sec in real time. Measurements start after 100 sec, defined as time 00∶00 (min∶sec). The mean position of each T cell is registered every 10 seconds and is used to calculate displacements, speeds and migration angles. Motility coefficients and persistence times were estimated from mean square displacement plots by fitting Frth's equation for a persistent random walk (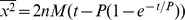, where 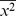 is the mean square displacement, 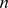 is the dimension of the space, 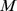 is the motility coefficient, 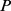 is the persistence time and 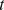 is the elapsed time period since the start of the trajectory) [40], [52] to the data using the software package R (freely available at http://www.r-project.org/). Interactions between T cells and DCs are registered every second and are considered contacts when they touch each other at at least one grid point.

## Supporting Information

Figure S1
**Chemoattraction increases the total number of T-DC contacts.** (**A, B**) The mean number of T cell contacts per DC for the DC-contact mechanism (**A**) and the no-desensitization mechanism (**B**) as a function of the strength of chemoattraction (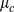) during 1-hour CPM simulations. Symbols represent means over 20 simulations, error bars represent standard error of the mean between simulations and colour codes in (**A**) represent the values for the recovery times used. Note that the difference with Fig. 3 in the main text is that here a renewed interaction between the same T-DC pair is counted as a new interaction, whereas this is not the case in the main text figure.(EPS)

Figure S2
**T cell scanning by DCs with desensitization upon contact with the chemokine gradient.** Unique number of T-DC contacts as a function of the strength of chemoattraction (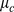) during one-hour CPM simulations for the gradient-contact desensitization mechanism. (**A**) Varying desensitization times, combined with a fixed recovery time of 5 min. (**B**) Varying recovery times, combined with a fixed desensitization time of 5 min. The threshold values along the vertical axes indicate the chemoattractant concentrations (in arbitrary units) at which a T cells starts its count-down to desensitization. Results are shown as means over 20 simulations, error bars represent standard error of mean between simulations, and colour codes denote the various desensitization or recovery periods used.(EPS)

Figure S3
**Finding a rare antigen in 2D.** (**A**) Percentage of 1000 cells that establish contact with a single cognate DC producing chemokine. For each value of 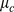, ten simulations were performed per desensitization mechanism (each circle represents the outcome of one simulation, while crosses represent the averages of these simulations per chemotaxis strength). The desensitization time for the DC-contact mechanism was set to 15 min. (**B**) The time it took for T cells to find the chemoattracting DC as a function of the starting distance from that DC. Circles represent measurements from individual T cells and lines denote the mean search time per distance bin of 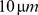. Note that the means at large distances are based on few datapoints only.(EPS)

Video S1 **T cell motion in absence of chemotaxis.** Fragment of a simulation, with T cells without recent contact with a DC (blue), T cells who had a recent contact (yellow), DCs (red), reticular network (green), and the ‘LN capsule’ at the top (cyan). T cells leave at the boundary at the bottom.
(MP4)

Video S2
**T cell motion with chemotaxis.** Fragment of a simulation with a strength of chemoattraction of 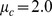, with sensitive T cells (blue), insensitive T cells (yellow), DCs (red), reticular network (green), and the ‘LN capsule’ at the top (cyan). T cells leave at the boundary at the bottom.(MP4)
